# Corrigendum: Sport Karate and the Pursuit of Wellness: A Participant Observation Study of a *Dojo* in Scotland

**DOI:** 10.3389/fsoc.2021.748612

**Published:** 2021-08-24

**Authors:** Fabiana Cristina Turelli, Carlos María Tejero-González, Alexandre Fernandez Vaz, David Kirk

**Affiliations:** ^1^ PhD Student, Universidad Autónoma de Madrid and University of Strathclyde, Madrid, Spain; ^2^ Professor of Departamento de Educación Física, Deporte y Motricidad Humana, Universidad Autónoma de Madrid, Madrid, Spain; ^3^ Professor of Estudos Especializados em Educação, Universidade Federal de Santa Catarina, Florianopolis, Brazil; ^4^ Professor of Education, University of Strathclyde and Honorary Professor of Human Movement Studies, The University of Queensland, Brisbane, QLD, Australia

**Keywords:** wellness/wellbeing, karate-sport, martial art, ethnography, participant observation

## Introduction

If we take into account the increasingly intense pace of life that society adopts, the health problems that emerge are numerous, and stress is one of the leaders on the list (Tapley, 2007; Fuller and Lloyd, 2020). To deal with these problems, there appears to be a growing social consensus that the practice of physical activities, including martial arts, is an extremely pertinent option (Rios et al., 2018). Martial arts are traditionally practised as an alternative to dealing with violence, often as a form of self-defence (McCaughey, 1997; McCaughey, 1998; Hollander, 2004; Matthews and Channon, 2016) or as a way of finding spiritual and bodily harmony and serenity. In the latter case, some martial arts are often associated with meditative and spiritual practices, coming to be related to a religious basis (Brown et al., 2009; Mainland, 2010; Cushing, 2013; Contiero et al., 2018; Kumpf, 2018).

The martial modalities best known for promoting a state of inner peace and balance are Tai Chi Chuan, Aikido, Kendo, and from a certain point of view, Yoga (Mainland, 2010; Wang et al., 2013; Kumpf, 2018). The concept of wellness has been repeatedly associated with these practices, since its followers seem to find physical and psychological relaxation through them and a certain inner connection associated with their spirituality (Draxler et al., 2010; Cushing, 2013; Turelli Thume et al., 2018). In this context of the achievement of wellness, karate possesses, as we will show, certain peculiar characteristics. On the one hand, karate is a martial art that has this traditional aspect, that is, the philosophical and meditative character (Kumpf, 2018; Cavalcante and Potiguar, 2019). However, on the other hand, it has also developed as a sport, with an explicit focus on competition, on the effectiveness of techniques, on the rules to achieve the best results and, ultimately, it has now achieved the status of Olympic sport (www.wkf.net, accessed on April 10, 2020; see also Chan, 2000 and Bowman et al., 2010 for further depth). *Karate-do* is a martial art of Japanese origin that translates as the *path of empty hands*. It was developed in Okinawa so that the people, devoid of fighting weapons, could defend themselves using their own bodies (Krug, 2001; Lautert et al., 2004). Over time, karate’s goal became the improvement of character through mental and physical discipline obtained with arduous training (Cavalcante and Potiguar, 2019). It seems that the transition to the West was responsible for the conversion of the martial art into sport (Macedo, 2006; see also James and Jones, 1982 on the introduction of karate specifically in Britain and Maclean, 2015 about karate in Scotland). In different practice environments today, the tradition of martial art is mixed with modern aspects of sport.

Our study reported here is focused on a *dojo* ^1^ in which its practitioners mixed or united two initially and apparently opposite things. They practiced a strictly sporting form of karate which stimulated extreme excitement and fatigue with the aim of achieving wellness. This was in contrast to forms of karate and other martial arts that seek to achieve a meditative state for wellness. More than anything, they wanted to reach different levels of being well through controlled aggression, the intensity of spilling something contained internally. Perhaps we can say that it is a question of achieving some balance through an apparently unbalanced path. We say this because we participated in this process by immersing the first author of this paper in the field, in the practices and in the social life of the club. Thus, we were able to live intimately the experience of a certain imbalance, euphoria and, perhaps, ecstasy, which is generated in the midst of the stimuli to be aggressive, the strident *kiais*,^2^ the sweat, the panting, the burning muscles and the punches and kicks. Somehow, it seems that these *karateka* ^3^ need more than to recharge their energies, they need to empty themselves through exhaustion. Finding pleasure is perhaps achieved by punishing the body.

We did an eight-month immersion in a karate club in the Glasgow area, Scotland. Initially, our goal was to see how this conversion from karate to Olympic sport was impacting the club’s practitioners. Such a club was dedicated to the competitive scenario, having affiliation with the World Karate Federation, the body responsible for karate approved as an Olympic sport. However, to our surprise, the practitioners appeared quite nonchalant about karate’s new Olympic sport status and the data indicated another main motivation for them to dedicate themselves to karate. They focused on achieving personal wellness goals, in a very broad sense, using intense karate training. As we said, the *dojo* is dedicated to the competitive scenario, so the training focuses on preparing for competitions, in an intense way.

We know that a certain pedagogy of pain and suffering (Vaz, 2000; Vaz et al., 2005) or even the pedagogy inherent in bodily fights (Turelli, 2008) is not exclusive to karate. Fights, in general, forge the body of the fighter (Wacquant, 2002). And there are several sports that, by promoting a certain degree of agony and punishment of the body, also generate some satisfaction in their practitioners. This is found in athletics, ballet and karate in a comparative way, for example (Gonçalves et al., 2012), in addition to performing in other modalities such as CrossFit, judo, among others (Howe, 2004; Parry, 2006; Green, 2011). We understand that karate fits among sports that inflict a certain degree of pain and suffering on the body, but not danger or any risk to life, as can occur in extreme sports (Brymer and Mackenzie, 2017). Perhaps they all generate adrenaline or even other elements in common, however, they definitely seem to be different categories. There are painful sports and dangerous sports as different things (Brymer and Schweitzer, 2017). We will not enter the field of extreme sports because we understand that karate is not contained there.

However, what emerges as an element of distinction in this investigated group is the high level of elaboration of the criteria for the submission of their bodies there, in that environment. In other words, it is not simply a matter of massacring one’s own body to exhaustion on the dirty mat–which can be quite recurrent in the sport martial context, even having a peculiar unpleasant smell that characterizes the environment–it is necessary to find beauty in practice. It is not just a matter of being effective and decisive in blows, but it is necessary to master the art of control. Furthermore, more than achieving technical perfection, it is expected that the technique is dosed in combination with an aggressive attitude. Perhaps we can say that the use of the body until exhaustion is no longer a matter of just the infringed requirement, for this group, even if scientifically calculated. In addition to science, art is added here, with a number of criteria required by practitioners to choose karate as a practice that leads them to wellness, which is such a complete and even complex concept.

We consider that it is of great relevance to take into account the significance that this particular group attributed to martial practices. Because this meaning responds to the needs of the members of this *dojo*, respecting the peculiarities of karate in Scotland and perhaps in the United Kingdom, but also possibly may represent other *dojos*, although not studied yet. Thus, the purpose of this paper is to present the representations that *karateka* of the *dojo* investigated by us make of sportive karate as a resource to achieve wellness. In addition, our methodology involved the ethnographic immersion of the first author of the study in the investigated context, participating in training, competitions and, to a large extent, in the life of the *dojo*. Before entering the field of representations that karate acquires for the achievement of wellness, however, we will briefly focus on the presentation of conceptual differences between wellbeing and wellness, to clarify the concept to which we refer here.

## Wellbeing and Wellness

Wellbeing has long been associated with quality of life. However, after a series of arguments from different researchers on the topic, Dodge et al. (2012) concluded that quality of life is just a dimension of wellbeing among other important ones. For the authors (Dodge et al., 2012), wellbeing needs to take into account the physical, psychological and social aspects of life. Thorburn (2015), on studying the implications of wellbeing for education, performed an important review of the theoretical constructs associated with wellbeing. He presented different authors considering varied elements in the composition of the concept, and worked on the idea of authenticity, for example, to achieve fulfilment and life-satisfaction. He reported how teachers felt insecure about working “values” with students to develop wellbeing. And so, along with other arguments, Thorburn (2015 p. 658) exposes how education systems in New Zealand and Scotland, for example, have “a lack of consensus across disciplines and sectors about what wellbeing means.”

On the other hand, the Global Wellness Institute defines wellness as “the active pursuit of activities, choices and lifestyles that lead to a state of holistic health” (https://globalwellnessinstitute.org/, accessed in February 01, 2020). An important element of this definition is to actively position oneself in the search for this state, in intentions, actions and choices, and not passively wait for something to happen. Wellness is an individual search, however, it relates to the environment where a person lives, physically, socially and culturally.

There is undeniably some confusion between the terms wellness and wellbeing. However, wellbeing–and a certain ability to feel happy–seems to be something that is found as a subsection of wellness (Kirsten et al., 2009). In other words, wellness encompasses six dimensions: physical, mental, emotional, spiritual, social, and environmental. These dimensions range from very concrete things, such as nutrition, exercise and social interaction, to finding meaning in life (https://globalwellnessinstitute.org/, accessed in February 01, 2020).

Thus, although the *karateka* in this study sometimes use the word wellbeing–and even just “fitness”– we conclude that they are referring to the complete and broad state of wellness. As noted above, we recognize that there is a confusion between the terms and, following Kirsten et al. (2009), we decided to adopt the term wellness because we understand that it is the broadest and most comprehensive of them. The *karateka* referred not to a simplistic conception, but rather an elaborate one. Actually, the place where the *dojo* is located is a municipal leisure centre, a place that offers activities that aim to promote health associated with leisure and pleasure. This place seems to be about reaching the state of psychosomatic harmony without making use of painful treatments and, on the contrary, providing enjoyment. Leisure and pleasure are closely related to this context. So it is absolutely understandable that people go to such centres to be healthy, but also to feel good, to enjoy themselves, make friends, have coffee together, share banter humour^4^ and laugh (Mainland, 2010).

For the *karateka*, the state they seek to achieve ranges from basic physical skills and feeling good about their own bodies, to a noble sense of accomplishment with their own lives. The good feelings that the hormones produced by practicing karate as a sport generates is an evident fact. However, the *karateka* also wanted to meet subtler elements, which reveals a high demand and expectation that they have in relation to martial practices. In order to find the state of deep satisfaction, some people aimed to deal well with fear, perceive beauty, develop an aggressive attitude and also be in control. That is, they referred to a very complete concept of wellness. They enjoyed their lives in the *dojo*, they found their leisure and their peculiar dose of pleasure there. We wrote in the introduction this is peculiar because practising karate as a sport is certainly not a common or simple way, at face value, of finding pleasure. On the contrary, it is quite complex, wide and comprehensive. For this reason, in order to cover all aspects that may be contained in the sense that they intended to express, we will adopt in this paper the term wellness as mentioned, unless it is a direct quote from the *karateka*’s interviews where they used wellbeing or fitness.

## Methodology

We conducted this study in Glasgow, Scotland for 8 months, from April to December 2019. The period comprised two phases. The first of these, lasting 4 months, was the approach period. It consisted of observations, participating in karate training three times a week, totalling 6 h a week, as well as participation in competitions. The first author kept a field diary of these observations and also wrote a weekly blog reflecting on her experience, which served as the focus of a weekly debrief process with the last author, who commented on and discussed the blogs with the first author. The second phase, while the participant observations and competitions continued, was mainly characterized by the first author carrying out semi-structured interviews with members of the *dojo*. In total, there were ten interviews with nine *dojo karateka*. We requested the tenth interview with the *sensei* ^5^ to clarify and deepen some points that emerged in the initial analysis of the data, meaning that the *sensei* was interviewed twice. The interviews lasted an average of 1 h and followed a standard guide drawn up with open questions, taking into account points that emerged in the written reports from the participant observations of the first author. All interviews followed the same guide, but, as is normal, some points had more or less relevance according to the interpretation of each *karateka*. We will use in this paper the data from nine interviews due their relevance to the topic, since one of the interviewees did not emphasize the importance of wellness or even fitness as the other eight interviewees did. As we will highlight later, the theme of wellness was something that emerged from the field, that is, we were not looking specifically for it. However, when pointed out by eight of the nine respondents, it evidently attracted our attention.

### Trustworthiness of Data Analysis

Trustworthiness was achieved by the following processes. Reports were prepared for 65 training sessions carried out in the period and also for the four competitions attended by the first author. In addition, we deepened our reflections on what we saw and experienced in 33 weekly blogs. The blogs were a space for reflection, open and of free choice, which were written weekly by the first author, and then shared with the last author and discussed in meetings between them. This is a very relevant point to mention, since these meetings served to triangulate the analysis of data. This process carried out with blogs, field notes, and debrief meetings, allowed the elaboration of the ideas and new concepts to be generated. In this paper specifically, however, we will focus on the data collected through the interviews. All were recorded and transcribed using the free software *Otter*, version 2.1.4–1,518, as support. Once the transcription was completed, we proceed to categorizing the data. The data presented here are the part of the analysis that refers to the motivations of the practitioners, why they dedicated themselves to karate.

### Participants

The criteria used for the selection of the interviewees was especially their degree of integration in the *dojo*, their very frequent participation in training, and a certain influence that they seemed to have in the group as a whole. We interviewed two *sensei*, both men, and six students, three of whom were women. Among the *sensei*, one of them is the owner of the *dojo* and responsible for the karate training of all the others, including the second *sensei*. He is dedicated to being an instructor at the club he created in Glasgow 14 years ago, and before that he was instructing elsewhere, doing so for more than 20 years in total. The second *sensei* has been training in karate for 10 years. Among the students, there were people who had degrees in karate from fourth *kyu* to first *dan* ^6^, with a minimum of 2 years of training in this *dojo*. Among those interviewed, four people had experiences in different martial arts or different karate *dojos* before choosing this *dojo*. Four of the eight participated in competitions, one had already retired from competition, one was terrified of competing and two were considering the possibility of starting to compete. Their ages range from 31 to 55 years, with an average of 41 years.

These members of the study have very varied personal backgrounds, which seems to be a characteristic of the group as a whole. Among them there was a PhD, four people with university degrees in different areas, one person studying at undergraduate level and two people without university degrees. Only one of the interviewees identified as “foreign,” even though this person had lived in Glasgow for 13 years. The rest are British, although two people were born in or spent parts of their lives in other countries. The competitive landscape in which these people move and even the *dojo* as a whole is, however, Scottish. In other words, only the main *sensei* has dedicated himself in the past to participating in international competitions.

These people represent the age group of the adults that make up the club. In addition to this age group, many children and adolescents are present in the *dojo*. However, the people that could be in the 20–30s, for some reason unknown to us, are not present in this *dojo*. Unfortunately, this data did not attract our attention during the period of immersion in the field, which would have led us to its timely investigation. Anyway, we find that this is the reality of this club and we are not generalizing it or forcing its extension to all other *dojos* that are dedicated to the practice of sports karate. Some results may be transferable, in fact, but further investigation would be necessary to prove or deny such transferability.

### Ethics

We obtained written authorization from the owner/s*ensei* of the *dojo* to proceed with the research. All the interviews we did had an oral and written presentation of the research objectives, its justification, the researchers’ data and that the study was part of a doctoral project. All respondents were left with a copy of the document signed by us and we also had copies signed by them giving consent. The study was approved by the Ethics Committee of the Universidad Autónoma de Madrid. In order to preserve the participants’ identities, we will not mention their names at any time, just using random letters to designate their interviews’ quotes.

### Monist and Dualist View

We want to point out that a very recurrent and dichotomous view of body and mind exists between martial artists of different modalities (Rios et al., 2018; Ongpoy et al., 2017; Tadesse, 2017; Burt and Butler, 2011). Perhaps we can suppose that in some cases it is a philosophical heritage with a dualistic orientation. However, in other cases, there could be a tendency to use the dualistic language while at the same time trying to express a monist experience. We consider this possibility once we know that we learn from the body, that it is not the mere executor of commands (Contiero et al., 2018). But the fact is that looking at body and mind separately has become something widely accepted and, more than that, almost consensual. So, the view that *dojo* practitioners present is not a rarity, but a psycho-social reflex. However, our proposal is more holistic, a monist view. We highlight our position and, taking this into account, we will keep the literal quotations with the dualist language that sometimes was used by the participants.

### Authorship, I and We

Before moving on to the narrative of the investigative process, we clarify that the parts that directly refer to the collected data will be presented in first person singular. We decided on this way of reporting since the immersion in the field took place with the participation of the first author of the study, as we already said. The intimate character that the reports acquire with writing in the singular seems to be maintained with greater fidelity, from our point of view.

We consider it important to point out some things in relation to the immersion in the field made by the first author, who will speak in the first person, since the researcher participating in the environment becomes the main instrument of data generation. The fact of conducting research from inside, as a participant in the *dojo* as well as a researcher, is a factor that has the potential to distinguish our study from others. Because it is not just talking about something that is seen from the outside, but something that is felt on the skin, with which one has intimate contact. It is not a matter of presenting the views of those involved only, but also of including the experience itself, lived, felt, attested by self-submission to what others also do.

Perhaps the first thing to say is that the investigated environment was both known and unknown to the first author. I have known karate for a long time, and this joined me to the group as a *karateka* companion. However, this was in my first contact with the country (Scotland), with a new language for me and a different culture, elements that I found strange. Perhaps something similar happened with Delamont (2009) when studying capoeira in the United Kingdom. I was a strange stranger, playing with words a little, using karate as a familiar language. Whenever anthropologists write about ethnography, they speak of the importance of making the everyday object of study strange. Undeniably, I went through this process. And even the object of study, karate, familiar to me, became strange, because it was adapted to the local culture. For example the names of the blows, traditionally spoken in Japanese, were in this *dojo* translated into English.

Besides that, participant observation is very rich and complex. While observing others, their actions and reactions, it is also necessary to be attentive to one’s own actions and reactions. I sought to do everything naturally and at the same time with intention. Naturally so that the experience is authentic and not simulated, but with care so that the experience is not totally spontaneous to the point of forgetting that you are investigating, not *going native*, as risk in anthropology. And with intentionality because of thoughtful interaction, nimbly thought out, can trigger expressions, manifestations, behaviours of extreme richness for research. Anyway, I see that there is no ready recipe for this. Just being “on the inside” seems to predispose people to cooperate and share what they know since they suppose I live the same way as them. I believe that my posture naturally followed this line, even though some things turned out to be truly strange for me^7^.

I am aware that my position in the field and my social condition may have had some influence on the material collected and its analysis. It was not the first time that I had the experience of arriving at a *dojo* as a novice and experienced at the same time. Novice because I just arrived, and experienced because I am a black belt. In this situation, everyone watches you and makes their assessments. In addition, I was a foreigner in the environment, needing help with the language often, something I was never denied and seemed to challenge and amuse *karateka*. They also reacted respectfully to my status as a researcher and, in time, absorbed it and, perhaps, forgot it. Finally, my social condition as a woman, perhaps more than impacting the field, continually directed my gaze towards reading the situations, recording the most relevant to me and being present in the data analysis. I consider that my position as a woman cannot be abandoned. And I think that if the analysis were performed by the men in the *dojo*, it would take other directions. However, the theme we developed in this article is not controversial and came as a novelty to me.

Amidst the scenery of some strange elements was the conception and search of *karateka* for elaborated wellness. I admit that it was the first time that I looked at the sport, in its hard version, from this point of view. Since finding something like serenity and completeness amid punches, kicks and *kiais* of the sport, and not in Zen practise that martial art could assume, was not something that I expected to find.

### Discovering the Wellness Construct

“Your mind and your body are doing the same thing at the same time, in the moment. (…) You are just absolutely in the moment. And everything … is just … your body is just responding really, really well. Has a huge beauty to that, mentally, it makes you feel very, very well; and physically makes you feel well. It’s a feeling of absolute wellbeing, I think.” (“V,” Interview 9, October 11, 2019).

This quote from one of the *karateka* helped to indicate the path, a little confusing at first, to what the practitioners were referring to, to what they sought with their practice. Because many people started their practice seeking to be fit, whether it was losing weight, keeping in shape, or managing stress. So they said that when they feel healthy, they had found a pleasant physical, psychological or mental state. Besides, they were happy with the relationships they established in the context of the *dojo*. All of this, for them, seemed to be their self-perception of being fit. They appreciated this condition, because in karate this conception of being fit adds to the acquisition of skills by making them feel more confident. Perhaps I can say that they used to receive important help in this direction from the main *sensei*. He was remarkably skilled in different aspects of karate. Usually he did not put himself as extreme authority in karate or even other subjects, he adopted an open posture and, at first sight, appeared democratic in his running of the *dojo*. However, the fact that he was/is undeniably skilled makes everyone respect him and naturally gives him the position of highest authority, the first place in the hierarchy.

*Sensei* often made comments to students as feedback on their performance and such comments were often positive. That is, he, from the height of his magnificent performance, recognized that the students were progressing, that they were advancing very well. From my own experience, this generates a sense of wellness with oneself. People crave it. And I think it contributes to building self-confidence and even self-esteem. This all translates into the language used in the *dojo* as an aggressive attitude. It is necessary to be confident of oneself to present the posture that does not hesitate, to have attitude and determination.

I think this set of elements conforms to the meaning of wellness for this group. In this great “umbrella” called wellness there is space for different facets of karate. The first facet is the fitness itself related to losing weight or keeping in shape. There are four other subcategories. One is about beauty as a broad concept, containing aesthetics, embodied aesthetics as well, but not limited to it. A second is about fear, presenting itself under different aspects. A third is the aggressive attitude, an important and desired posture among the *karateka*. Then control, fourth, other central concept in this environment.

## Findings and Discussion

### “I Want to be Fit Enough to be Prepared to Deal With Whatever Comes Along”—The Importance of Fitness.

Regarding fitness related to losing weight or keeping in shape, all respondents attached some value to this theme.

“I wanted to get fit, and I plan to get fit alongside doing karate and put my own fitness efforts in.” (“Q,” Interview 2, September 24, 2019).

When I began to hear the discourse about fitness repeatedly in interviews, I started to wonder about its importance. Whether it would be someone’s obsession, a cultural issue, or if there would be some strong public policy related to health, nutrition and exercise. Anyway, I noticed that there was a consensus about the use of this term fitness, that is, everyone considered that seeking fitness is appropriate. In fact, after carrying out a systematic review of the health benefits of adults who practice hard martial arts, Rios et al. (2018) highlighted the reports of positive effects for physical and mental health. Initially I understood that *karateka* referred only to these aspects when using the term.

For three of the interviewees, becoming fit was the reason for starting karate and two of those talked about a striking change in their bodies for weight loss. They said that it is important to continue with the practice so as not to change again in a negative way, gaining weight in this case.

“Now I feel as if it’s in my head, if I don’t go I’m going to put all the weight I’ve lost. I’ve lost six stones. Maybe more. I was really, really overweight. (…) I’m terrified my fitness is going to go, not so much the weight, it’s more of the fitness. So, and I feel so much better, you know.” (“N,” Interview 7, October 03, 2019).

Five people are added to this observation of shape improvement or maintenance, even saying that they are now better than in their 20s.

“I’m physically better now than in my 20s. Stronger and more capable. It’s addictive!” (“Z,” Interview 1, September 23, 2019).

“I am able to have the same performance now that I had in my 22 years old.” (“X,” Interview 3, September 25, 2019).

“I’m good, I move as a 20-year-old.” (“H,” Interview 6, October 03, 2019).

“I don’t feel it, but the fact is that I am 55!” (“W,” Interview 8, October 06, 2019).

They also attached value to the fact that in addition to being well physically, they enjoyed this type of exercise more than in activities such as weight training or gymnastics, generally more monotone activities. For Draxler et al. (2010) the main benefits of practicing martial arts are observed physically. In this *dojo* though, practitioners also developed a social life and seemed to feel active at different levels, prepared to deal with different situations that arise in their lives.

“It’s a great way to keep your mind and body active and fit.” (“X,” Interview 3, September 25, 2019).

“I want to be fit enough to be prepared to deal with whatever comes along.” (“Q,” Interview 2, September 24, 2019).

To be fit seemed to be a state that contains movement, displacement, some agility, strength and flexibility. But more than that, it relates to some degree of self-defence ability, which is developed through the individual appropriation of karate techniques. I found it extremely interesting to note that as this ability develops, people became self-satisfied with their overall performance. And then, something like a cycle is created, where people find a certain accomplishment and keep practicing what generates in them pleasure, besides meeting initially-set goals for physical fitness.

Perhaps we can say that the initial objective gains more depth, that is, it could no longer be enough, after a certain time, just achieve a desired physical shape or keep fit. More complex concepts began to appear, such as beauty, not only in the visible forms of movement, but in the perception and sensation that beauty produces in the whole body.

### “I Think I Find Quite Beautiful, I Find the Moves Quite Beautiful, I Like the Way my Body Feels Doing it”—Beauty in Punches and Kicks?

It is hard to find beauty in the midst of violence. Perhaps it is not impossible when one thinks for example of nature in action, storms and other natural phenomena. However, in my opinion as a *karateka*, physical violence is devoid of beauty. I am not able to appreciate self-defence aesthetically, with its hard, dry blows in the pursuit of effectiveness. It is different from the aggressive attitude, discussed below. Already in those blows, although they are punches and kicks that could previously seem violent, when they are performed with technical precision, fluid, controlled and fast, as required by the sportive aspect of karate, I can identify tremendous beauty. (Allen, 2015). asks in his book *Striking Beauty: A Philosophical Look at the Asian Martial Arts* “where is the beauty in something so vested in violence?” In order to find a balance between beauty and violence he says that “movement tends to become aesthetically interesting as it becomes fluid, flowing, efficient, visibly, energetic, and seemingly effortless” (2015 p. 158). To some extent “V” also identifies this feature:

“I can see some beauty (in kumite^8^) when I watch two people who are very good, hitting together.” (“V,” Interview 9, October 11, 2019).

This view is in line with what *karateka* present especially when they talked about how they feel performing the movements. The state of wellness was attained as the experience of sensations occurring in the body.

“I love the discipline of it (kata^9^) and I love, I think I find quite beautiful, I find the moves quite beautiful, I like the way my body feels doing it.” (“V,” Interview 9, October 11, 2019).

I believe this feature of karate relates to the lived experience of the body, that which is already embodied (Downey, 2010; Velija et al., 2012; Downey et al., 2014), which comes to fruition and produces pleasure when put into practice. When something new is started, some degree of discomfort is usually experienced. But when it becomes practiced, it belongs to the body in some way, it becomes comfortable to perform, and more than that, the experience of performing such movements generates delight. As the body is shaped in the karate environment, movements practiced repetitiously become internalised and therefore comfortable to perform.

“It’s how to introduce repetition without being too boring. But at the same time that becomes your body, your body. It’s like practicing piano and I play the same thing over and over again. And because it’s your … in your fingers.” (“Z,” Interview 1, September 23, 2019).

When the body is able to work autonomously, and sometimes a little automatically, the *karateka* can try other things, develop new actions. That is, it is possible to be aware of other elements and explore them more. Schmidt (2004) did a revaluation about his own schema theory first published in 1975, related to motor skill learning. The proprioceptors in the muscles and kinesthesis permits the *karateka* a degree of freedom and they can at the same time as acting, create opportunities, explore possibilities, observe the opponent, think ahead. And that undeniably produces self-satisfaction, wellness. Allen (2013. p. 251) says that “true speed and power come with eloquence, when the many movements become one beat, one corporeal melody beautifully performed–if, that is, something so violent can be beautiful at all.”

“Kumite is creative, very creative. You take the techniques and then you have to have the speed, and the meta agility to put all together.” (“V,” Interview 9, October 11, 2019).

Thus, it seems that beauty is not so much seen, that is, it is not perceived with the commonly used sense for that, vision. It, the beauty, is, indeed, felt. It is an aesthetic experience (Kirk, 1996; Maivorsdotter and Lundvall, 2009; Maivorsdotter and Quennerstedt, 2012) that includes some degree of satisfaction with one’s own body. It is the experience of flow (Csikszentmihalyi, 2000), a certain possession of one’s own body, in the sense that there is power over it, which works and responds according to the commands it receives/feels exactly in time. It is the body present and flowing, without deviating from what it needs to do. The *karateka* is present, concentrated, attentive, active.

“Your mind and your body are doing the same thing at the same time, in the moment. (…) You are just absolutely in the moment. And everything … is just … your body is just responding really, really well. Has a huge beauty to that.” (“V,” Interview 9, October 11, 2019).

Perhaps I can synthesize the perception of beauty in this environment, not for all participants, at least consciously, but for some of them, as a state and a feeling (Van Deventer et al., 2007). It is possible to see and appreciate beauty in perfectly performed techniques, for example, but it is also possible to feel. At times, I heard participants telling me that this or that experience was “addictive.” It impacted me at first. But now I think I can understand a little better. Maybe they only refer to this state that they find in different forms that karate can take, more or less appreciated according to the taste of each person. Notwithstanding they get addicted to the state, the feeling, the sensations. And they want more. They wanted to feel more of it.

Wellness is found in something beautiful, pleasant. But the way to reach it can be hard. The next theme is about this, about a certain discomfort with which it is necessary to learn to deal and live with.

### “Be Comfortable in Being Uncomfortable’—How to Embrace Fear.

There are different forms of fear present among *karateka* in this context. A very strong form is the fear of failure. This is probably central here. Failure can be presented as defeat in competition, as not performing to one’s potential, including in training. There is a fear of taking risks and receiving penalty points from the opponent. This is not about fear of being beaten by the opponent, but of being caught, that she or he scores on you.

“In competition is so little room for error. And not always the better person wins.” (“X,” Interview 3, September 25, 2019).

There is also a certain fear of the sensations that come with competition, the suffering of when there is a desire to escape, the discomfort, the pressure. (Sagar and Stoeber, 2009). establish a relationship between the fear of failure and perfectionism and also say that these concepts are usually associated with shame and embarrassment.

“I am quite good and people know that I’m quite good. That puts pressure on me. I don’t know. But I’m good.” (“H,” Interview 6, October 03, 2019).

Some thoughts are also feared. So it turns out that *karateka* her/himself feeds the fear.

“If you put your mind into it, you know, you can’t. You can’t think too much. Just think about like dancing now, when you dance you don’t really start thinking, “I’m gonna move my body here and there.” Yes, just kind of internalize, okay, this is a pattern you have to do. Just relaxing your body and then just start doing this. Yes. So in those cases, yes, like the body working alone without the mind.” (“Z,” Interview 1, September 23, 2019).

A second form of fear is associated with injury. This type of fear most often appears in people a little older. This is the fear of heavy blows and a certain request for control, which I approach in the last section. There is a certain fear of pain apparently, perhaps because pain has previously been experienced.

“I would like just 5 min in the end (of kumite), not more, to avoid an injury.” (“W,” Interview 8, October 06, 2019).

(De Pero et al., 2013) point to the relationship between anxiety and injuries in a study they conducted with 14 Italian gymnasts. It is interesting to observe this, because “W” told me that he always feels anxious on his way to the *dojo*. Likewise, he fears *kumite*, because with contact with the opponent in the fight he thinks he can get hurt. So he would like just 5 min of *kumite* at the end of training, being completely warm.

The next type of fear is related to that, training itself and what one can experience in it, the pain that the training sessions can bring. The *sensei* used the expression “sweet pain” a lot. It is supposedly sweet and should be appreciated because it brings progress to people’s performance. Nevertheless, the *karateka* kept coming to practice. They feared and respected at the same time other *karateka* who caused them pain and progress, ultimately *sensei*. Perhaps there is some kind of love-hate relationship with the practice and among people, in this environment.

“The pain comes according “X”‘s mood.” (“H,” Interview 6, October 03, 2019).

““X” is very bad. He doesn’t know when stop.” (“H,” Interview 6, October 03, 2019).

Practitioners recognized the *sensei*’s skills, respected and admired him. However, they repeatedly pointed out, in moments of relaxation, how the *sensei* enjoys hard training and, supposedly, seeing his students suffering in training. The *sensei* says he does that for the sake of *karateka*. That is why “H” said that the measure or the moment to stop is unknown to the instructor, or that according to his mood the training hurts more or less. Anyway, even though *karateka* suffer during the execution of routines, they seem to like and want more of it.

In that context, *karateka* were guided to taste and enjoy the pain. Analogously it was being said that pain and fear must be accepted. Fear and pain should not be repudiated. (Spencer, 2012). presents an interesting study on this topic, especially related to pain in mixed martial arts. The author comments that there is an intimate relationship between the way athletes express their feelings of pain and masculinity. Surely this also occurs among *karateka*, who needed to learn to deal with pain and fear and live with them. Perhaps over time these elements may decrease, but possibly they will not be completely overcome.

“Be comfortable in being uncomfortable. Know you will be uncomfortable and just be okay with that.” (“V,” Interview 9, October 11, 2019).

As a researcher, I consider this to be an excellent phrase, while as a *karateka* I hate it. I think this because I have very clear and present sensations and feelings that make me uncomfortable, especially fear in some situations. I hate this feeling. But I always face it again–and this seems to be exactly the same for the other *karateka* in the *dojo–*. I don’t get over it, it’s always there. Nor does it seem to diminish. I see how easy it is to understand and agree with that phrase, but I also see it as almost an attack simply to be okay with the discomfort. The discomfort is intense, continuous, visceral. And yet we get back to that. (Bolelli, 2008). works very well with the idea of using martial arts as a tool to lose fear in life, especially fear from different forms of conflict. He says that “through practice of the fighting arts, the martial artist stares his (or her) own fears in the eyes. He (or she) challenges them every time when facing an opponent. Every fight is a battle against our own limits and weaknesses.” (p. 5) “Z” captured this in her comment that.

“Karate is experience of facing your fears, being uncomfortable.” (“Z,” Interview 1, September 23, 2019).

Where is the relationship between feelings of fear and wellness? This is a very interesting question and exposes a little of the peculiarity of this group that finds leisure and pleasure in uncomfortable activities. It seems that *karateka* use their experiences on the mat as a laboratory for life. They feel stronger in the *dojo* for overcoming or at least facing the situations that present themselves and this strengthens them in a way, generates in them a certain feeling of comfort, however controversial it may seem, of pleasure and even of a certain superiority. In this context, they learn elements that shape the posture they choose for their lives. Another of these elements is the aggressive attitude.

### “I’ve Learned How (…) to be Aggressive in the Right Way. Aggressive Without Losing Your Temper”—The Aggressive Attitude as a Posture in Life.

Possessing the right level of aggressive attitude is a serious goal among *karateka*. In fact, I think I can say that it is better to have something like an abundance of that attitude than to lack it.

“Not at all, I’m not aggressive. As I’m … “X” tells me I say “sorry” too much. I always say “sorry.” “Sorry.” He says the next time I say “sorry,” he used to say the class … “The next time “N” says “sorry,” we’re doing 10 push ups”.” (“N,” Interview 7, October 03, 2019).

In general understanding with karate, it is a posture, a position to face life. According to the reading the *karateka* do, it is not possible to present a *kata* without power or to enter the *kumite* as a coward, and outside the *dojo*, in ordinary life, to be an extremely brave person. The posture that people adopt in the *dojo* is one that they have in their everyday lives, in short. So everyone longs to exhibit this attitude-laden posture. It is frustrating not to own it, as “W” said:

“I’m aggressive, but I’m not over the top. Definitely, I’m not over the top. (…) I just don’t have greatest offensive techniques, so it’s almost frustrating, is like “okay, you hit me, I can’t do the same back,” you know what the mean?” (“W,” Interview 8, October 06, 2019).

Some people naturally and innately have a certain level of aggression (Lim et al., 2010). Other people need to develop it, to cultivate it, build in themselves. In the current view in the *dojo*, if one does not have this posture, unless there is a commitment to build it, there is a gap, a lack, a void. The *sensei* commented in this respect that.

“Technique is pretty easy to fix; attitude is so difficult.” (“X,” Interview 10, November 01, 2019).

Countless times in interviews and training sessions *karateka* talk about this attitude as a kind of passion. This posture is identified with a strong and intense charge of energy, of a kind of enthusiasm, of passion in synthesis. That is an important word, passion. Adopting a passive posture is something that is to be avoided, repudiated in some cases and quietly despised.

“You cannot win on defensive.” (“Z,” Interview 1, September 23, 2019).

The active and intense posture is instead sought and praised (Daniels and Thornton, 2008). This is also emphasized in the competitive environment, which rewards this attitude. The introduction of *senshu* ^10^, for example, presents itself as a stimulus for athletes to attack. The defensive posture may, in a way, be punished. The points system is aimed at making the sport attractive to the public, certainly, and forces athletes to pursue an aggressive stance and attitude.

“My style is going to be more aggressive, because I think I am a bit more aggressive than probably I realize perhaps.” (“Z,” Interview 1, September 23, 2019).

It draws attention as “Z” presents hesitations in her speech: “I think,” “probably,” “perhaps.” The environment fosters the aggressive attitude and commonly people, at least those who remain, tend to adapt to the context. Thus, it is possible that they develop–or want to develop–what they did not initially bring, or even discover something that was a little hidden even for themselves and now can/should come up.

Attitude, posture, passion, are superior aspects even to the results in competitions. It is possible to lose and have great honour if everything was done intensely, giving yourself completely, without hesitation.

“I’m an attacker fighter. It’s about intimidating sometimes, I think.” (“H,” Interview 6, October 03, 2019).

Being unpredictable in the fight also seems to be quite positive, because it is an active attitude. There is no limit to strategies, you can use whatever you want if it helps to present the aggressive attitude.

“That’s the aim, to be predictably unpredictable.” (“X,” Interview 3, September 25, 2019).

“When I step on the mat and I look that person I just think terrible things. It makes me fight better. You don’t do that?” (“H,” Interview 6, October 03, 2019).

“H” asked me that question as if to say that such an attitude is the normal and correct one. I replied to him that I don’t have such thoughts and maybe that explains a little bit why I am able to see the environment really as strange often. However, it is essential to always maintain a certain degree of control. If the aggressive attitude, especially the aggression, goes beyond the tolerated level and becomes something like out of control, there is also some repudiation for that. Thus, it is necessary to be aggressive, but there is a right way to express it. A good *karateka* can never afford to lose one’s mind. As “K” put it succinctly.

“I’ve learned how to do that and how to be aggressive in the right way. Aggressive without losing your temper.” (“K,” Interview 4, September 26, 2019).

So, it is important to find the right balance between aggressive attitude and control, something very appreciated in the *dojo*. And reaching the exact measure or approaching it is something that delights practitioners. It is necessary to work to achieve this, to submit, and they do it, again because every time they see their goal, of someone strong, aggressive and controlled, like an archetype, such a vision stimulates and pleases them. They pay the price for this satisfying feeling. It means to say that the effort for wellness, as a goal and also in the small conquests of the way, is justified.

### “Control is a Massive Thing in Karate for me. You Know, the Ability to be Aggressive but Controlled”—The Superiority Given by Control.

Control is a fundamental criterion for competitions and also for a respected *karateka*. Developing control generates wellness because people feel capable to do karate well, they feel competent. According to (Madden, 1995), the perception of increased control makes people feel less vulnerable.

The rules in competition and in the *dojo* dictate that you need to score while respecting your opponent’s physical integrity. In competitions for juniors this criterion is more strictly enforced. Already for adults there is a measure between the control and the “touch,” that is, tapping a little, “marking the point.” Scoring the point objectively means hitting a little. It is not to hit with violence, but to hit with aggressive attitude, with determination. Especially in the abdominal region this is allowed as it is mandatory to wear chest and rib protection under the *gi*.^11^ The abdominal themselves, on the other hand, is out of protection, and all of this can be “touched” if there is no effective defence. It is a region that is the responsibility of each athlete to keep trained and strong to withstand certain strikes. A *karateka* will only be penalized for hitting this region if she or he does so in an open manner with the intention of hurting. If the blows are on the head and face, the criteria are stricter and much more control is required.

Thus, there is contact and some apology for it. At times this is not so fair, that is, it is common in fights to use strong blows to intimidate the opponent. Even if you receive a penalty for this, the rules allow for a number of penalties, and sometimes the impact on your opponent may, in the opinion of some coaches, make up for it. For they may react with fear, retreat, be intimidated. Even in training the *sensei* emphasizes that it is necessary to “touch,” have some aggression. After all, this also needs to be trained.

“It doesn’t sound good, but that absolute manipulation on the mat, on the tatami. I’m going to manipulate you to where exactly I want you. (…) I call like a shepherd with the sheep. Sheep dog, “come on, in the corner.” I love that.” (“X”, Interview 10, November 01, 2019).

But sometimes people don´t react well. And this aspect is a bit intriguing because while *karateka* want to be physically challenged in training, they care very much not to receive blows. This seems to have a slightly offensive facet, the person who received the blow feels somewhat assaulted (Wojdat and Ossowski, 2019). As “Z” put it, “I don’t like the stress of being hurt.” (“Z,” Interview 1, September 23, 2019)

There are opinions that are contradictory. Because at the same time that it is necessary to fight with control, not to be exceeded, not to hurt the training companion, especially, one has to “touch.” “X” raised the question, “Why would somebody who doesn’t want to get hit come to karate?” (“X,” Interview 10, November 01, 2019).

And so people start to develop this ability to “hit a little,” something that is not contrary to the spirit of the sport. For many, it is not natural, like the aggressive attitude. In fact, this skill is part of the aggressive attitude that is often built in the *dojo*. For some, the idea of hitting another *karateka* clearly does not come naturally.

“I took a while to actually become comfortable with actually hitting somebody.” (“K,” Interview 4, September 26, 2019).

In fact, training with someone who never gets close enough to “score the point” on you doesn’t help you get better. You stop defending and the fight becomes something like a game between two nice people, but it is not mutually challenging. On the other hand, if the person does not allow you to approach for some kind of fear and so your blows are always far away, neither is this positive. Because your body does not have the actual experience of reaching the head or the area in focus. Everything becomes a joke. When you find a training mate who is willing to train closer to the reality of competition, it is highly valued. But it does require a measure of control, as I commented before. Aggressive attitude cannot become violence (Twemlow and Sacco, 1998). If there is loss of control, there is loss of respect. It is a sign of superiority not to use the same language or to pay in the same currency what is negligible. As “K” commented,

“I wouldn’t feel that I have to fight fire with fire.” (“K,” Interview 4, September 26, 2019).

On the contrary, there is great admiration for those *karateka* who are able to exert control of their blows. Practitioners want to train with those with the highest belts for two main reasons, supposedly that belts are able to challenge them to give their maximum, and they have enough control in general not to harm them.

“Control is a massive thing in karate for me. You know, the ability to be aggressive but controlled is so important.” (“K,” Interview 4, September 26, 2019).

“I’m looking up we’re you know, the higher belts and I’m thinking I wish I was in that group because it pushes me more you know, and I feel as a failure and more like I love going with “K” I love going with you, even with “Q”, you know.” (“N,” Interview 7, October 03, 2019).

Achieving the level of mastery of the technique necessary to develop exquisite control, situated between “touching” and teaching is something that challenges and attracts *karateka*. They aimed for a degree of challenge that will make them progress and everyone, without exception, has the view that being challenged is positive, everyone wants to go beyond their comfortable limit. The challenge of achieving control, something that few obtain with true mastery, is a differential that places its holder in a position of a certain symbolic superiority. This social place is, therefore, another exotic ingredient of wellness for this group.

## Conclusion

“It (karate) pushed me so hard and I love that feeling of being pushed in having to train harder and get better and all that type of thing.” (“K,” Interview 4, September 26, 2019).

In this paper we wanted to present the reasons why a Glasgow *karateka* group practice the sport. Thus, we relate hard-trained sportive karate to some unconventional conceptions that practitioners develop about wellness. In addition, we added to the two previous elements, karate-sport and wellness, the participant observation carried out by the first author. We highlight the conventional and typically accepted relationship between traditional martial arts and the meanings of wellness. Such a relationship occurs succinctly by the search and achievement for inner peace, harmony and balance through meditative, protocol following, philosophical and, in some cases, spiritualistic martial practices (Draxler et al., 2010; Mainland, 2010; Cushing, 2013; Wang et al., 2013; Kumpf, 2018). And we also present peculiar aspects of sportive karate that do not fit the well-known martial arts standards that usually promote wellness, but which for the investigated group, has shown to provide deep and quite elaborate levels of wellness.

Through thematic analysis, the data led us to identify the achievement of wellness by excess, excitement, tiredness, euphoria, fatigue, punishment of the body, challenge, (self) demand, discomfort and feeling of superiority. The *karateka* achieve wellness using body work, working on their physical shape, the first reading of fitness, as perhaps the most superficial level of their conception of wellness. This is something consensually important. Then they develop elaborate levels of this conception. The pleasure that gives them the appreciation of the beauty seen, but especially felt in themselves, when performing. They enjoyed the aesthetic experience from punches, kicks, *kiais*, carrying out complex and energetic techniques. They also lived their leisure experience when facing fear and seeking to accept the discomfort present in some moments. This decision to submit to the unpleasant builds, in their view, a strong posture for life. Also, it contributes to the solidification of this posture the development, whether by channelling something innate, awakening something that slept, or building something that does not exist, of the aggressive attitude. The possession of this attitude is a distinguishing factor (Bourdieu, 2007) among practitioners and gives them positions of a certain superiority in the internal hierarchy of the *dojo*, especially when accompanied by control. If control is an absent characteristic, it is possible to lose that position. However, if it is present, with all the challenge that control conquest entails, it guarantees extreme–perhaps cathartic (Hollanda, 2009)– sensations of wellness. Although we find in the literature a vast number of sports in which the punishment of the body as a sports pedagogy (Vaz, 2000) is also present, we identified in this group certain differences. Perhaps the main difference is the elaboration and possible refinement of the criteria adopted by the *dojo* to achieve wellness. Demanding and extremely strenuous elements are there, combined with tradition, hierarchy, discipline, creativity, beauty, aggressive attitude, control, discomfort, fear, flow. Elements that, if not combined, perhaps with something of science and art, would only be contradictions.

In this sense, this group showed us an atypical way to achieve wellness. They provoke us to think that they don’t want to feel full, filling in the gaps that people usually say they have, figuratively, internally. These *karateka* aim instead to empty themselves, *spending*/*using* themselves through practices, perhaps through a somewhat tortuous and impactful path. This is the sensation felt in the flesh itself when participating in the training and living the social life of the *dojo*. And this leads us to reflect on the importance and need for new investigations to see if this is repeated in other martial arts, if many more groups find wellness overflowing and experiencing situations of (self) confrontation in the same way. Was there, for example, some degree of sadism/masochism involved in these *karateka*’s experiences? Why do they need to find wellness through a hard and demanding path and do not feel served by the traditional meditative and relaxing? Why do they elaborate their practice in a peculiar way, combining apparently antagonistic elements? We therefore provisionally close this study, given the need for its continuity.

## Footnotes

1 Name in Japanese to the martial practice location.

2 *Kiai* is the scream accompanying the blows. It is understood as a *cry of power*, not just a cry from the vocal cords.

3 The term used for karate practitioners.

4 Category of traditional Scottish humour, similar to black humour. It can be quite heavy and, according to *karateka*, it can help to strengthen the psyche, making people not easily offended.

5 Is the graduated teacher, sometimes more than that, a kind of master.

6 *Kyu* and *dan* are the Japanese terms used to designate grades in karate. The coloured belts are the *kyus* and count down. When the first *kyu* is reached, the next degree is *dan*, the black belt. From then on, the count is increasing.

7 I have realized that many things that I perceive as strange in the countries where I am a foreigner or stranger, in this case Spain and the United Kingdom, have more similarities to each other than my country of origin, Brazil. They are similarities, not identical correspondences, but they still lead me to reflect that, perhaps, what is strangest is the culture of my country of origin.

8 *Kumite* is the fight itself within karate, structured by weight categories.

9 *Kata* is the Japanese name for the fight against an imaginary opponent. It is performed as a choreography of blows, in synthesis.

10 It is the *advantage* obtained by scoring the first point in *kumite*. If the fight ends in a draw, the athlete holding the *senshu* will be the winner.

11 The karate uniform, sometimes incorrectly called *kimono*.
